# Recapitulating muscle disease phenotypes with myotonic dystrophy 1 induced pluripotent stem cells: a tool for disease modeling and drug discovery

**DOI:** 10.1242/dmm.034728

**Published:** 2018-07-18

**Authors:** Ricardo Mondragon-Gonzalez, Rita C. R. Perlingeiro

**Affiliations:** 1 Lillehei Heart Institute, Department of Medicine, University of Minnesota, Minneapolis, MN 55455, USA; 2 Departamento de Genética y Biología Molecular, Centro de Investigación y de Estudios Avanzados del IPN (CINVESTAV-IPN), México City 07360, Mexico

**Keywords:** Myotonic dystrophy, Induced pluripotent stem (iPS) cells, Skeletal myogenesis, Muscular dystrophy, PAX7, RNA foci

## Abstract

Myotonic dystrophy 1 (DM1) is a multisystem disorder primarily affecting the central nervous system, heart and skeletal muscle. It is caused by an expansion of the CTG trinucleotide repeats in the 3′ untranslated region of the *DMPK* gene. Although patient myoblasts have been used for studying the disease *in vitro*, the invasiveness as well as the low accessibility to muscle biopsies motivate the development of alternative reliable myogenic models. Here, we established two DM1 induced pluripotent stem (iPS) cell lines from patient-derived fibroblasts and, using the PAX7 conditional expression system, differentiated these into myogenic progenitors and, subsequently, terminally differentiated myotubes. Both DM1 myogenic progenitors and myotubes were found to express the intranuclear RNA foci exhibiting sequestration of MBNL1. Moreover, we found the DM1-related mis-splicing, namely *BIN1* exon 11 in DM1 myotubes. We used this model to test a specific therapy, antisense oligonucleotide treatment, and found that this efficiently abolished RNA foci and rescued *BIN1* mis-splicing in DM1 iPS cell-derived myotubes. Together, our results demonstrate that myotubes derived from DM1 iPS cells recapitulate the critical molecular features of DM1 and are sensitive to antisense oligonucleotide treatment, confirming that these cells can be used for *in vitro* disease modeling and candidate drug testing or screening.

This article has an associated First Person interview with the first author of the paper.

## Abstract (precis)

**Summary:** This work provides proof of principle for the use of myotonic dystrophy 1 patient-specific induced pluripotent stem cells to model muscle pathology *in vitro* and in drug discovery.

## INTRODUCTION

Myotonic dystrophy 1 (DM1) is an autosomal dominant multisystemic disorder that causes myotonia and progressive muscle weakness and wasting. DM1 is the most common type of adult-onset muscular dystrophy worldwide (affecting 1 in 8000) (Harper, 2001), and is caused by a CTG triplet repeat expansion within the 3′ untranslated region (UTR) of the dystrophia myotonica protein kinase (*DMPK*) gene on chromosome 19 q13.3 (Mahadevan et al., 1992). Although the progression of the disease is variable among patients, in general, the severity of the symptoms is directly proportional to the length of the CTG repeat expansion and inversely proportional to the age of onset (Yum et al., 2017). Various molecular events related to the CTG repeat expansion have been associated with the disease phenotype (Klesert et al., 2000; Reddy et al., 1996; Ho et al., 2005), but the most relevant is a toxic RNA gain-of-function of the *DMPK* mutant transcripts (Mankodi et al., 2000). mRNAs containing expanded CUG repeats fold into extended stem-loop structures that form RNA foci (Taneja et al., 1995; Napierala and Krzyzosiak, 1997; Tian et al., 2000). These RNA foci are retained in the nucleus and interact with RNA binding proteins, such as Staufen1 (also known as STAU1), hnRNP H (also known as HNRNPH1) and members of the MBNL family (Ravel-Chapuis et al., 2012; Paul et al., 2006; Miller et al., 2000). Sequestration of the splicing factor MBNL1 by the RNA foci, which leads to splicing disruption of MBNL1 target genes, is the main molecular feature associated with DM1 skeletal muscle pathology (Meola and Cardani, 2015; Tang et al., 2012; Fugier et al., 2011). Yet, DM1 is a complex disease that remains to be fully understood.

Myoblasts obtained from patient muscle biopsies have been widely used to study DM1 and other muscular diseases *in vitro* (Arandel et al., 2017; Pantic et al., 2016). However, performing a biopsy is an invasive procedure and samples are usually not easy to access. Thus, it has been of major interest for the field to generate alternative myogenic models that can be reliably used for *in vitro* disease modeling and/or drug screening purposes. The reprogramming of somatic cells to a pluripotent state, in which they are known as induced pluripotent stem (iPS) cells, provides the possibility of differentiating patient-specific iPS cells into multiple lineages (Takahashi et al., 2007), including skeletal muscle (Darabi et al., 2012). Furthermore, iPS cells can be expanded indefinitely, which makes them a robust cell source that overcomes the limited expansion potential of patient-derived myoblasts or fibroblasts for high-throughput drug screening.

Reprogramming of DM1 patient-derived somatic cells to iPS cells has been previously described to study the central nervous system (Ueki et al., 2017; Yanovsky-Dagan et al., 2015; Xia et al., 2013; Du et al., 2013), but, to date, studies aiming to model the DM1 skeletal muscle pathology are still lacking. To fill this gap, here we reprogrammed DM1 patient fibroblasts into iPS cells, and evaluated whether differentiation of DM1 iPS cells into the myogenic lineage would recapitulate the molecular features of the disease. The results we show here demonstrate that this is the case, and that DM1 iPS cells represent a valuable model to study DM1 muscle pathogenesis.

## RESULTS

### Characterization of DM1 patient-derived fibroblasts and iPS reprogramming

**Fig. 1.**
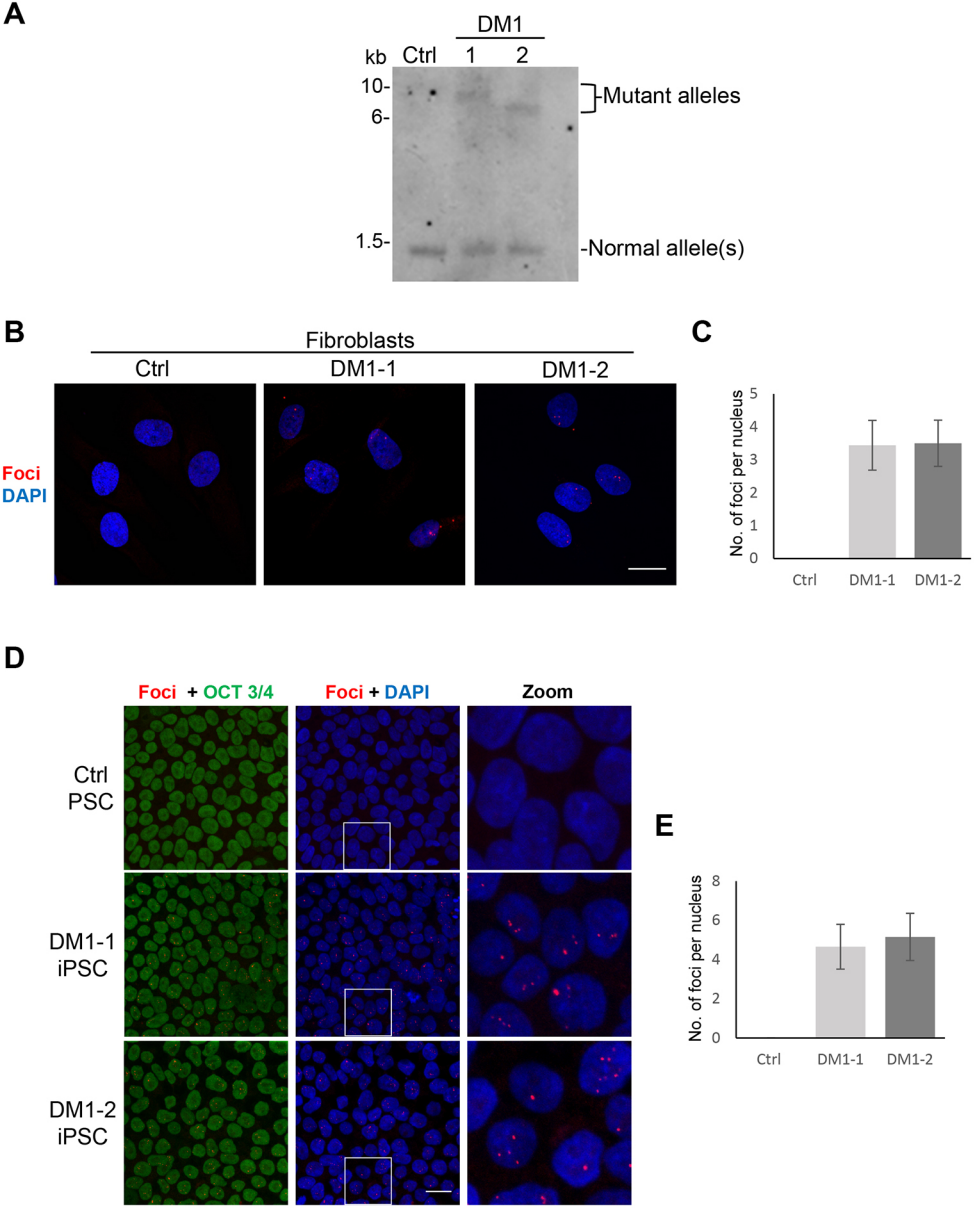
**Molecular characterization of DM1 patient-derived fibroblasts and reprogrammed DM1 iPS cells.** (A) Southern blot analysis using a digoxigenin-labeled probe binding to the 3′ UTR of the *DMPK* gene to determine the length of CTG repeats in fibroblast samples from two DM1 patients (referred to as DM1-1 and DM1-2). Fibroblasts from an unaffected individual were used as control. The DM1-1 sample showed an expansion of ∼2000 CTG repeats, whereas the DM1-2 sample contained ∼1500 CTG repeats. (B) Representative RNA-FISH images showing foci only in fibroblasts from DM1-1 and DM1-2 patients. A Cy3-labeled (CAG)_7_ probe was used to detect the foci (in red) and DAPI was used to stain nuclei (blue). Maximum projection of the *Z* sections is shown by confocal microscopy. Scale bar: 20 μm. (C) Quantification of foci (from B), represented as average number of foci per nucleus in 150 cells. Bars indicate s.d. from three independent experiments. (D) Representative images of RNA-FISH (red) coupled with immunostaining for the pluripotency marker Oct 3/4 (green) in DM1 iPS cells (iPSC) and control iPS cells (PSC). Maximum projection of the *Z* sections is shown by confocal microscopy. Scale bar: 20 μm. (E) Quantification of foci (from D), represented as average number of foci per nucleus in 150 cells. Bars indicate s.d. from three independent experiments.

As a first step in assessing the potential of patient-specific iPS cell-derived myogenic derivatives for the *in vitro* modeling of DM1, we reprogrammed skin fibroblast samples obtained from two diagnosed DM1 patients into iPS cells. Sample DM1-1 was obtained from a 35-year-old male patient bearing an expansion of 716 CTG repeats, whereas sample DM1-2 was obtained from an 18-year-old male patient with 473 CTG repeats, according to diagnosis in blood cells for both patients. The molecular features of DM1 were characterized in both fibroblast samples. Southern blot analysis showed an expansion of ∼2000 and ∼1500 CTG repeats in DM1-1 and DM1-2, respectively (Fig. 1A), which suggests mosaicism of the repeat length in somatic cells. Furthermore, fluorescent *in situ* hybridization analysis targeting ribonucleic acid molecules (RNA-FISH) using a Cy3-labeled (CAG)_7_ probe showed the presence of typical intranuclear RNA foci (Fig. 1B,C).

We then reprogrammed DM1-1 and DM1-2 fibroblasts using the Sendai virus (SeV) transduction approach. Three weeks after transduction, iPS cell colonies showing typical embryonic stem cell-like colony morphology were picked and expanded for ten passages (Fig. S1A). For each patient-specific iPS cell line, three clones were selected for pluripotency characterization. Gene expression analysis demonstrated that DM1 iPS cells display expression levels of the endogenous pluripotency factors *OCT3/4*, *SOX2* and *NANOG* similar to control embryonic stem cells (Fig. S1B). RNA levels for the reprogramming SeV were detected only as a faint band on day 7 after transduction but were absent in expanded iPS cells, as expected for this nonintegrating vector. Expression of the endogenous pluripotency factors was confirmed at the protein level (Fig. S1C). The pluripotent capabilities of DM1-1 and DM1-2 iPS cells were validated by their ability to develop teratomas upon their subcutaneous injection into immunodeficient mice (Fig. S1D). Importantly, no karyotypic abnormalities were found in generated DM1 iPS cells (Fig. S1E). Using RNA-FISH, we confirmed the expression of the intranuclear RNA foci in both DM1 iPS cell lines (Fig. 1D,E).

### Differentiation of DM1 iPS cells into skeletal myogenic progenitors

**Fig. 2.**
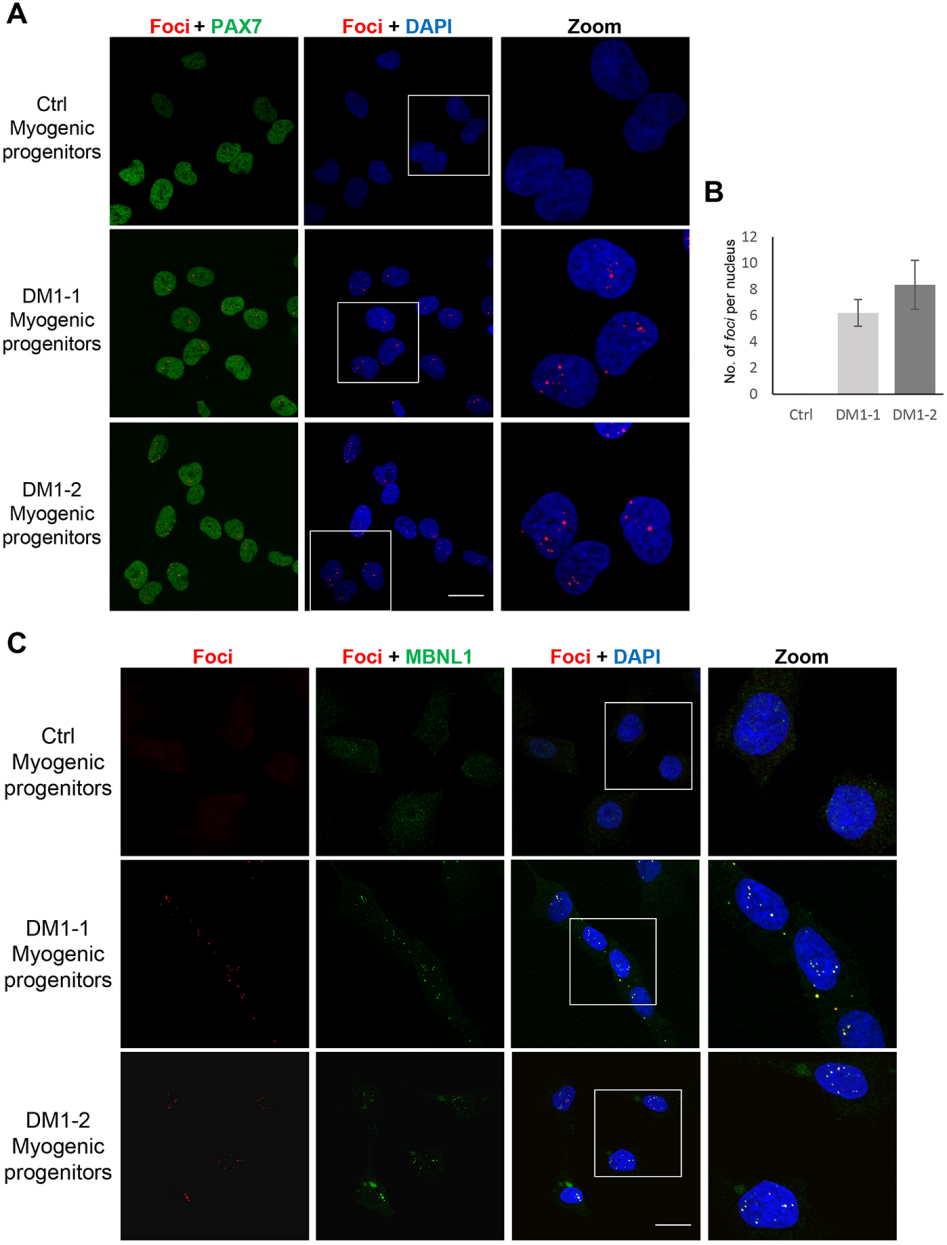
**Characterization of DM1 myogenic progenitors.** (A) Representative images showing RNA-FISH of foci (red) co-stained with the myogenic transcription factor PAX7 (green) in myogenic progenitors derived from control and DM1 iPS cells using confocal microscopy. Maximum projection of the *Z* sections is shown. Scale bar: 20 μm. (B) Quantification of foci (from A), represented as average number of foci per nuclei in 150 cells. Bars represent s.d. from three independent experiments. (C) Representative images showing RNA-FISH of foci (red) co-stained with the splicing factor MBNL1 (green) in myogenic progenitors derived from control and DM1 iPS cells. Mid *Z* section is shown. Scale bar: 20 μm.

Next, we differentiated DM1-1 and DM1-2 iPS cells towards the myogenic lineage using the doxycycline-inducible PAX7 system (iPAX7), as we have previously described (Darabi et al., 2012). To confirm whether DM1 iPS cell-derived patient-specific myogenic progenitors represent a valid model to study DM1-related features *in vitro*, we assessed the molecular phenotype of the disease in these cells. RNA-FISH analysis revealed the presence of intranuclear RNA foci in both DM1-1 and DM1-2 myogenic progenitors (Fig. 2A). The average number of RNA foci per nucleus was higher in DM1 myogenic progenitors than in fibroblasts and iPS cells for both patient-specific cell lines (Fig. 2B). As discussed above, another key molecular feature of DM1 is the intranuclear sequestration of MBNL1 by the RNA foci. To test whether DM1 iPS cell-derived myogenic progenitors recapitulate this process, DM1-1 and DM1-2 iPS cell-derived myogenic progenitors were evaluated for MBNL1 expression. We performed RNA-FISH followed by immunostaining with an antibody against MBNL1 in the myogenic progenitors. In control myogenic progenitors, we observed a diffused distribution of MBNL1 throughout the nucleus and cytoplasm, whereas DM1-1 and DM1-2 cells showed a pattern of staining that colocalized with the RNA foci, confirming MBNL1 sequestration in DM1 myogenic progenitors (Fig. 2C). We also observed RNA foci in the cytoplasm of these cells, in agreement with previous studies involving other affected DM1 cell types (Pettersson et al., 2014; Dansithong et al., 2008).

### Terminal differentiation of DM1 iPS cell-derived myogenic progenitors into myotubes

**Fig. 3.**
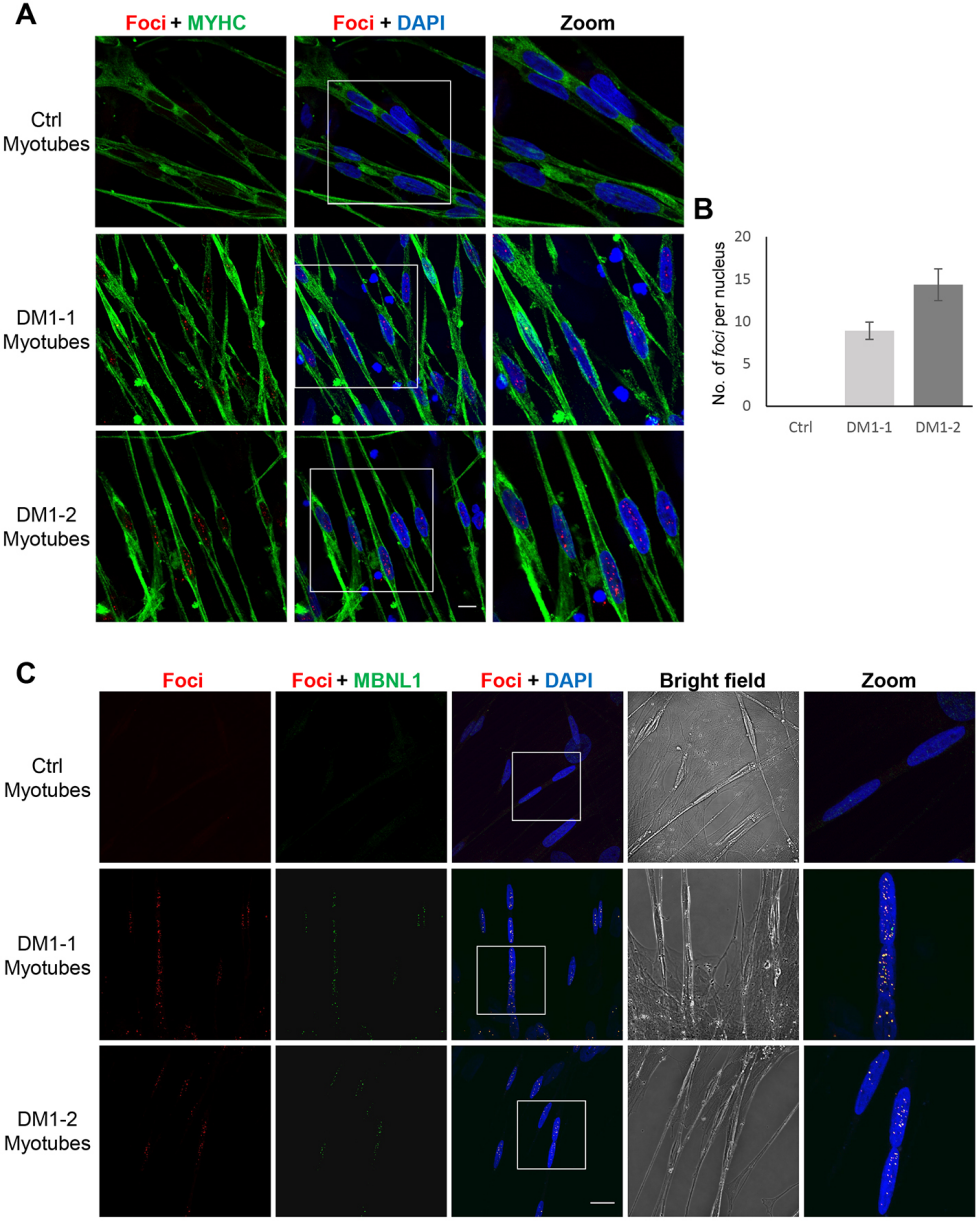
**Terminal differentiation of DM1 patient-specific iPS cell-derived myogenic progenitors into myotubes.** (A) Representative images showing RNA-FISH of foci (red) co-stained with MYHC (green) in control- and DM1-derived myotubes. Confocal microscopy shows maximum projection of the *Z* sections. Scale bar: 10 μm. (B) Quantification of foci (from A), represented as average number of foci per nuclei in 150 cells. Bars represent s.d. from three independent experiments. (C) Representative images of RNA-FISH (red) coupled with immunostaining of the splicing factor MBNL1 (green) in control, DM1-1 or DM1-2 myotubes, analyzed by confocal microscopy. Mid *Z* section is shown. Scale bar: 20 μm.

DM1-1 and DM1-2 iPS cell-derived myogenic progenitors were subsequently differentiated into myotubes expressing myosin heavy chain (MYHC), a marker of myogenic terminal differentiation, by culturing them to confluency, and then switching to a low nutrient medium (Fig. S2). RNA-FISH analysis of DM1-1 and DM1-2 myotubes showed the presence of intranuclear RNA foci (Fig. 3A). The number of foci in the myotubes was higher compared with the cell stages previously evaluated, particularly for DM1-2, in which we consistently found ∼14 foci per nucleus (Fig. 3B). Next, we analyzed the distribution of MBNL1 in DM1 myotubes and observed colocalization between MBNL1 and the RNA foci, therefore confirming the sequestration of this splicing factor (Fig. 3C).

### Reversal of DM1 molecular phenotype by antisense oligonucleotide treatment of DM1 iPS cell-derived myotubes

**Fig. 4.**
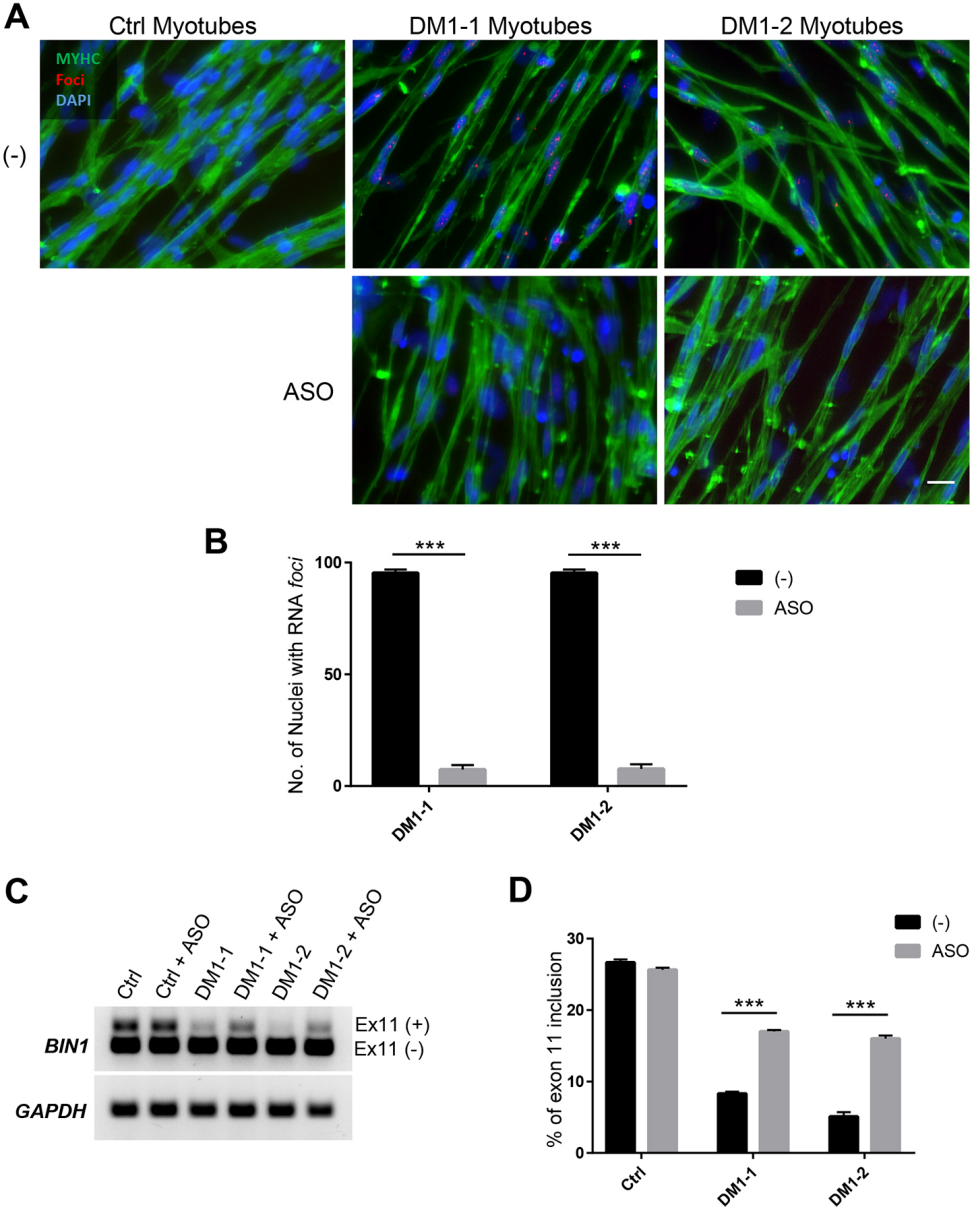
**Antisense oligonucleotide treatment reverses the molecular phenotype of DM1 iPS cell-derived myotubes.** (A) Representative images showing RNA-FISH of foci (red) coupled with immunostaining of MYHC (green) following 2′-OMe-PT-(CAG)7 antisense oligonucleotide treatment on DM1-1 or DM1-2 myotubes. Scale bar: 20 μm. (B) Number of nuclei showing RNA foci in three independent experiments (*n*=100). Data are mean±s.e.m. Comparisons were performed using the Mann–Whitney test. (C) Reverse transcription polymerase chain reaction (RT-PCR) analysis of *BIN1* exon 11 following 2′-OMe-PT-(CAG)7 antisense oligonucleotide treatment in control, DM1-1 or DM1-2 differentiated myotubes. (D) Percentage of *BIN1* exon 11 inclusion (from C) from three independent replicates. Data are mean±s.e.m. Comparisons were performed using the Mann–Whitney test. ***P<0.001.

The generation of DM1 iPS cell-derived myotubes provides the possibility of using these cells as an alternative to primary myoblasts for drug screening purposes. Therefore, we evaluated the effectiveness of treating DM1-1 and DM1-2 myotubes with 2′-OMe-PT-(CAG)7 antisense oligonucleotides (ASOs), which have been shown to abolish the RNA foci in myotubes differentiated from primary myoblasts (Mulders et al., 2009). One day after ASO treatment, we observed a significant reduction in the number of nuclei containing RNA foci in DM1-1 and DM1-2 myotubes (Fig. 4A,B). Subsequently, we assessed whether this reduction was sufficient to rescue the mis-splicing of *BIN1* exon 11, which has been related to myopathy and T tubule alterations, and was also found to be among the genes with higher splicing disruption in DM1 (Thomas et al., 2017; Fugier et al., 2011). We observed that upon ASO treatment of DM1-1 and DM1-2 myotubes, there was a significant rescue in the inclusion of *BIN1* exon 11, which correlated with the reduction of RNA foci (Fig. 4C,D). Of note, ASO treatment had no effect on the ability of DM1-1 and DM1-2 myogenic progenitors to differentiate into myotubes (Fig. S3A,B).

Overall, our results demonstrate that myogenic progenitors and myotubes differentiated from patient-specific DM1 iPS cells through the iPAX7-EB protocol display the key molecular features of DM1, such as intranuclear RNA foci, MBNL1 sequestration and subsequent splicing disruption. Hence, these cells represent a myogenic model that can be used as an alternative to primary myoblasts for studying the disease pathogenesis and/or drug screening purposes.

## DISCUSSION

Patient-derived myoblasts have been widely used for *in vitro* modeling of muscular diseases. However, expansion of muscle primary cells is limited by senescence and their terminal differentiation capabilities decrease upon passaging. Although establishing patient-specific immortalized myoblasts might overcome some of these hurdles, these have been modified to alter their cell cycle, and thus this aspect of cell physiology is abnormal. The reprogramming of human somatic cells into iPS cells by Yamanaka and colleagues (Takahashi et al., 2007) emerged as a promising tool to recapitulate muscle diseases in the Petri dish because these cells can be expanded indefinitely and are able to differentiate into several tissues, including skeletal muscle.

To date, several protocols have been established to promote the myogenic differentiation of iPS cells. One of the main advantages of our method based on the conditional expression of PAX7 is the generation of myogenic progenitors that can be robustly expanded *in vitro* (Darabi et al., 2012), as opposed to protocols based on MYOD (also known as MYOD1) induction, which give rise directly to more differentiated muscle cells and, accordingly, with limited proliferation ability (Tanaka et al., 2013). Furthermore, iPAX7 myogenic progenitors can be frozen/thawed by conventional methods and still efficiently differentiate into myotubes, which allows for the generation of large stocks of cells from the same preparation for further experiments. This feature is highly relevant for a myogenic model as it makes it a suitable source for high-throughput drug screening.

In this study, we differentiated DM1 patient-specific iPS cells into the myogenic lineage (Darabi and Perlingeiro, 2016) to determine whether these cells could recapitulate the main molecular events of DM1, and therefore be considered as a valuable alternative myogenic model of the disease. DM1 patient-specific iPS cells efficiently differentiated into myogenic progenitors able to terminally differentiate into MYHC^+^ myotubes. We found that DM1 iPS cell-derived myotubes display typical expression of intranuclear RNA foci along with sequestration of MBNL1, which is the main molecular event associated with the DM1 phenotype. This was further corroborated by identifying the splicing disruption of *BIN1*, a gene for which mis-splicing has been related to DM1 myopathy (Fugier et al., 2011). An important aspect to be evaluated in a model is its ability to validate previously tested drugs. In this regard, DM1 iPS cell-derived myotubes treated with ASOs showed a significant decrease in RNA foci along with significant rescue of *BIN1* exon 11 splicing.

An important aspect to be taken in consideration when studying DM1 *in vitro* and *in vivo* is the instability of the CTG expansions as these can expand or contract depending on the cell type or upon cell passaging *in vitro* (Musova et al., 2009; Nakamori et al., 2011; Monckton et al., 1995; Wong et al., 1995). Two recent publications making use of DM1 iPS cells have focused on this feature, and both concluded that despite increased instability of the repeats during the reprogramming of fibroblasts into iPS cells, this is minimized upon differentiation of DM1 iPS cells into specific lineages (Ueki et al., 2017; Du et al., 2013).

Taken together, we demonstrate the efficient differentiation of two newly reprogrammed DM1 patient-specific iPS cell lines into skeletal myogenic progenitors and subsequent myotube derivatives, which faithfully recapitulate key molecular events of DM1, making them suitable for *in vitro* disease studies and drug testing.

## MATERIALS AND METHODS

### Patient samples

De-identified cryopreserved skin fibroblasts from two diagnosed DM1 patients (DM1-1 and DM1-2) were obtained through the Paul and Sheila Wellstone Muscular Dystrophy Center at the University of Minnesota, according to procedures approved by the Institutional Review Board of the University of Minnesota. Cells were expanded in high-glucose Dulbecco's modified Eagle medium (DMEM) containing 10% fetal bovine serum (FBS), 1% GlutaMax, 1% penicillin-streptomycin, 1% sodium pyruvate and 1% nonessential amino acids under standard culture conditions.

### Reprogramming

DM1 fibroblasts were reprogrammed into iPS cells using a CytoTune™-iPS 2.0 Sendai Reprogramming Kit (Thermo Fisher Scientific) using feeder-free conditions, according to the manufacturer's instructions. Three to four weeks following the transduction of pluripotency factors, iPS cell colonies were picked, transferred to fresh dishes and expanded for ten passages to eliminate the nonintegrative SeV from the cultures. DM1 iPS cells were passaged with ReLeSR™ (STEMCELL Technologies) and cultured on Matrigel-coated dishes using mTeSR™1 medium (STEMCELL Technologies). For each patient-derived line (DM1-1 and DM1-2), three clones showing classic pluripotent stem cell colony morphology were used for pluripotency characterization, and one clone was used for subsequent studies.

### Teratoma studies

Animal experiments were carried out according to protocols approved by the University of Minnesota Institutional Animal Care and Use Committee. iPS cells were injected at 1.5×10^6^ in the quadriceps of 8-week-old male NOD-scid IL2Rg^null^ (NSG–Jackson Laboratory) mice. Before injection, cells were resuspended in 1:1 solution DMEM-F12 and Matrigel (final volume including cells, 65 µl).

### Myogenic differentiation

DM1-1 and DM1-2 iPS cells were transduced with a doxycycline-inducible PAX7 system (iPAX7) and differentiated towards the myogenic lineage as previously described (Darabi and Perlingeiro, 2016). DM1 iPAX7-iPS cells were dissociated with Accumax (Innovative Cell Technologies) and 1×10^6^ cells were cultured in a 60 mm Petri dish using mTeSR^TM^1 medium supplemented with 10 µM Y-27632 (ROCK inhibitor) and incubated on a shaker (day 0). On day 2, the medium was replaced with embryoid body (EB) differentiation medium [15% FBS, 10% horse serum, 1% KnockOut Serum Replacement™ (KOSR), 1% GlutaMax, 1% penicillin-streptomycin, 50 µg/ml ascorbid acid and 4.5 mM monothioglycerol in Iscove's modified Dulbecco's medium (IMDM)] supplemented with 10 µM Y-27632 and 10 µM CHIR990217 (GSK3 inhibitor). Following incubation of EBs in suspension for 3 days, the medium was replaced with fresh EB differentiation medium containing 10 µM Y-2763 to withdraw GSK3 inhibitor treatment. On day 7, EBs were collected and plated on gelatin-coated flasks to promote their adhesion and expansion as a monolayer using EB differentiation medium supplemented with 10 ng/ml human basic fibroblast growth factor (bFGF). On day 10, the medium was replaced with fresh EB differentiation medium supplemented with 10 ng/ml human bFGF+1 µg/ml doxycycline to promote PAX7-GFP expression. On day 14, GFP^+^ cells (PAX7^+^, myogenic progenitors) were sorted and expanded on gelatin-coated flasks using the same medium. DM1 or control iPS cell-derived myogenic progenitors were terminally differentiated into myotubes by growing them to confluency and then switching to terminal differentiation medium (20% KOSR, 1% GlutaMax, 1% penicillin-streptomycin in KnockOut™ DMEM). Myotube characterization was performed after 5 days of terminal differentiation. Throughout the study, we used a previously established and validated human iPAX7-iPS cell line (Darabi et al., 2012) as a nondisease control.

### Southern blotting

To determine the approximate length of the CTG triplet repeat in the DM1 fibroblasts samples, Southern blotting was performed. Briefly, genomic DNA (gDNA) was isolated from DM1-1, DM1-2 and control cells using the PureLink™ Genomic DNA Mini Kit (Invitrogen). Purified gDNA was digested with *Bam*HI, which generates a fragment of ∼1.4 kb from the 3′ region of the *DMPK* gene considering a normal allele containing 20 CTG repeats. Digested gDNA was run in a 1% agarose gel, denatured (1.5 M NaCl, 0.5 M NaOH), neutralized (1.5 M NaCl, 0.5 M Tris-HCl, pH 7.5), blotted to a positively charged nylon membrane by capillary transfer with 20× saline-sodium citrate (SSC) buffer, and fixed to the membrane by UV-crosslinking. To detect the *Bam*HI-digested fragment spanning the CTG repeats, a probe was design using the following primers (Fwd: 5′-TCCCCAACCTCGATTCCCCTC-3′; Rev: 5′-GGCCACCAACCCAATGCAGC-3′). Labeling of the probe and detection were performed with a DIG High Prime DNA Labeling and Detection Starter Kit II (Roche).

### RNA-FISH and immunofluorescence

To detect the intranuclear RNA foci, cells grown on coverslips were fixed with 4% paraformaldehyde for 10 min, permeabilized with 0.3% Triton X-100 for 15 min, and incubated with pre-hybridization buffer solution [2× SSC and 30% formamide in diethyl pyrocarbonate (DEPC) water] for 10 min at room temperature (RT). Cells were then incubated with hybridization solution [2× SSC, 30% formamide, 0.02% bovine serum albumin (BSA), 2 mM ribonucleoside vanadyl complex, 66 µg/ml yeast transfer RNA and 0.1 ng/μl of a Cy3-labeled (CAG)_7_ probe in DEPC water] for 2 h at 37°C in a humid chamber. Cells were washed twice with pre-hybridization buffer solution for 7 min at 42°C and two more times with 1× SSC in DEPC water for 5 min at RT. At this point, samples were either mounted on glass slides with ProLong™ Gold antifade reagent with 4′,6-diamidino-2-phenylindole (DAPI) (Invitrogen) or processed for immunostaining. For the latter, samples were blocked with 3% BSA in phosphate-buffered saline (PBS) for 30 min and incubated overnight with the appropriate primary antibody at 4°C. The following day, cells were washed three times with PBS for 5 min and incubated with the appropriate secondary antibody for 1 h at RT. Cells were washed again three times with PBS for 5 min and mounted on glass slides as described above. Samples were analyzed by confocal microscopy (Nikon NiE C2 upright confocal microscope).

The following antibodies were used for immunofluorescence: anti-OCT3/4 (C-10, Santa Cruz Biotechnology; 1:50), anti-SOX2 (Y-17, Santa Cruz Biotechnology; 1:50), anti-NANOG (H-2, Santa Cruz Biotechnology; 1:50), anti-PAX7 [PAX7, Developmental Studies Hybridoma Bank (DSHB); 1:50], anti-MBNL1 (3A4, Santa Cruz Biotechnology; 1:75), anti-MYHC (MF 20, DSHB; 1:100), Alexa Fluor 488 goat anti-Mouse IgG (Invitrogen; 1:500), Alexa Fluor 555 goat anti-Mouse IgG (Invitrogen; 1:500).

### RT-PCR

Samples were collected with TRIzol™ Reagent (Invitrogen) and RNA was purified using a Direct-zol™ RNA Miniprep Plus Kit (Zymo Research) following the manufacturer's instructions. Purified RNA was quantified with NanoDrop 2000 (Thermo Fisher Scientific) and retrotranscribed using a SuperScript VILO cDNA Synthesis Kit (Invitrogen) following the manufacturer's instructions. Complementary DNA (cDNA) was used as template for PCR using GoTaq Flexi DNA polymerase (Promega). Previously reported primers were used for pluripotency characterization of DM1 iPS cells (Chen et al., 2013) and for splicing analysis of *BIN1* exon 11 in DM1 iPS cell-derived myotubes (Fugier et al., 2011).

### Antisense oligonucleotide treatment

DM1 iPS cell-derived myogenic progenitors were seeded onto gelatin-coated plates for terminal differentiation. On day 4 (after switching to terminal differentiation medium), myotubes were transfected with 2 µg/ml 2′-OMe-PT-(CAG)7 antisense oligonucleotides using RNAi Max transfection reagent as previously described (Arandel et al., 2017). Twenty-four hours after treatment, cells were processed for RNA-FISH or RT-PCR analysis. To test the effect of ASO treatment on myotube differentiation, DM1-1 and DM1-2 iPS cell-derived myogenic progenitors were transfected with 2′-OMe-PT-(CAG)7 antisense oligonucleotides 1 day before switching to terminal differentiation medium, using the protocol described above. After 5 days in terminal differentiation conditions, cells were fixed and processed for MYHC immunofluorescence. Plates were imaged using a Cytation™ 3 Cell Imaging Multi-Mode Reader (BioTek), and images were analyzed by quantifying the ratio of percentage MYHC area to percentage DAPI area using Fiji (Schindelin et al., 2012).

### Statistical analysis

Statistical comparisons were performed using the Mann–Whitney test in Prism 7 software (GraphPad). *P*-values <0.05 were considered significant.

## Supplementary Material

Supplementary information
