# Supplementary material for: Recapitulating muscle disease phenotypes with myotonic dystrophy 1 induced pluripotent stem cells: a tool for disease modeling and drug discovery
Source: Dis Model Mech. 2018 Jul 18;11(7):dmm034728. doi: 10.1242/dmm.034728 (PMC6078411; doi:10.1242/dmm.034728)
Supplement: Supplementary information [file dmm-11-034728-s1.pdf]

FIRST PERSON

First person – Ricardo Mondragon-Gonzalez

First Person is a series of interviews with the first authors of a selection of papers published in Disease Models & Mechanisms, helping early-career researchers promote themselves alongside their papers. Ricardo Mondragon-Gonzalez is first author on 'Recapitulating muscle disease phenotypes with myotonic dystrophy 1 induced pluripotent stem cells: a tool for disease modeling and drug discovery', published in DMM. Ricardo is a PhD student in the lab of Bulmaro Cisneros at CINVESTAV, Mexico City, Mexico. He conducted the work in this article while on a collaborative visit to Rita Perlingeiro's lab at the University of Minnesota, Minneapolis, USA, where he investigated gene and cell therapies as approaches for treating skeletal muscle diseases.

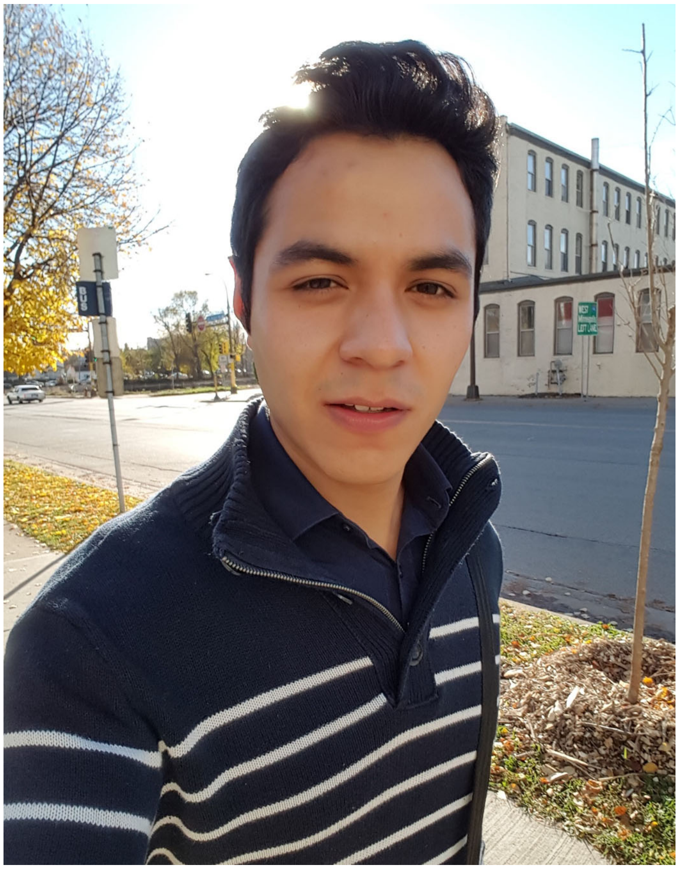

Ricardo Mondragon-Gonzalez

How would you explain the main findings of your paper to non-scientific family and friends?

Myotonic dystrophy type 1 (DM1) is the most common muscular disease in adults worldwide. Unfortunately, as for other muscular dystrophies, it has no cure. In order to develop potential strategies for the treatment of the disease, it is necessary to generate cellular models that allow us to study and understand the mechanisms underlying the phenotype of the disease and that can be used as a platform for high-throughput drug screening. We obtained skin cells from DM1 patients and reprogrammed them to a pluripotent stem cell stage (known as iPSC), which are cells with the potential to differentiate into different tissues. Using a protocol previously developed by our laboratory, we successfully differentiated these patient-derived DM1 iPSCs to skeletal muscle cells with the main molecular features of the disease. This iPSC-derived model will allow further research into the underlying causes of DM1 and represents a valuable tool for drug-screening assays.

What are the potential implications of these results for your field of research?

The DM1 myogenic progenitors that we have generated from patient-derived iPSCs are a valuable tool for *in vitro* disease modelling and drug-screening purposes in the myogenic context of the disease.

What are the main advantages and drawbacks of the model system you have used as it relates to the disease you are investigating?

> **"iPSC-derived myogenic differentiation is a field that is constantly being updated and further research is needed to better understand the full potential of this model."**

Patient-derived myoblasts have been widely used for *in vitro* studies of muscular dystrophies, including DM1. However, collecting primary myoblasts involves an invasive procedure and the availability of samples is usually limited. Furthermore, primary cell lines undergo senescence, which limits the expansion potential of this cell model. DM1 iPSC-derived myogenic models overcome those limitations as iPSCs have been described with unlimited proliferation capabilities. Furthermore, there is an increasing number of patient-derived iPSC lines being generated, which provides the possibility to study the disease in different backgrounds. Nonetheless, we acknowledge that iPSC-derived myogenic differentiation is a field that is constantly being updated and further research is needed to better understand the full potential of this model.

What has surprised you the most while conducting your research?

The identification of the molecular phenotype of DM1 (expression of intranuclear RNA foci) at different cellular stages (fibroblasts, iPSCs, myogenic progenitors and terminally differentiated myogenic cells) but with different abundance of expression in each stage is interesting as it could be related to the variability observed in the severity of the symptoms and the organs and tissues affected among patients.

Describe what you think is the most significant challenge impacting your research at this time and how will this be addressed over the next 10 years?

As DM1 is a hereditary disease with no cure, the most significant challenge is to find a potential therapy or treatment able to improve

Ricardo Mondragon-Gonzalez's contact details: Lillehei Heart Institute, Department of Medicine, University of Minnesota, Minneapolis, MN 55455, USA.
E-mail: mondr028@umn.edu

the quality of life of DM1 patients. However, increasing studies of gene therapy, cell therapy and drug screening have shown promising results that will continue to evolve over the next decade to hopefully find an efficient therapeutic approach.

"I believe that worldwide there is a lack of stable job opportunities for early-career scientists."

What changes do you think could improve the professional lives of early-career scientists?

I believe that worldwide there is a lack of stable job opportunities for early-career scientists. While postdoctoral positions offer an income and the opportunity to expand research experience, these are temporary. I think it is important to promote the need to increase job opportunities for early-career scientists.

What's next for you?

I feel attracted to skeletal muscle research and how new technologies (gene editing, cell reprogramming) are transforming and improving the approaches that we use to understand this fascinating field. Thus, I am interested in developing my scientific career in that direction.

Reference

Mondragon-Gonzalez, R. and Perlingeiro, R. C. R. (2018). Recapitulating muscle disease phenotypes with myotonic dystrophy 1 induced pluripotent stem cells: a tool for disease modeling and drug discovery. *Dis. Model. Mech.* **11**: dmm034728.
